# Bio-Fortified Pork Cracklings with UVB LED Tailored Content of Vitamin D_3_

**DOI:** 10.3390/foods11050726

**Published:** 2022-03-01

**Authors:** Petra Ložnjak Švarc, Marzia Rahimi, Jesper Tønnesen, Dennis Dan Corell, Paul Michael Petersen, Grethe Hyldig, Jette Jakobsen

**Affiliations:** 1 Research Group for Bioactives—Analysis and Application, National Food Institute, Technical University of Denmark, Kemitorvet, 2800 Kongens Lyngby, Denmark; petlon@food.dtu.dk (P.L.Š.); zendagi_jawid@yahoo.com (M.R.); 2100cph@gmail.com (J.T.); grhy@food.dtu.dk (G.H.); 2 Department of Photonics Engineering, Diode Lasers and LED Systems, Technical University of Denmark, Frederiksborgvej 399, 4000 Roskilde, Denmark; ddco@fotonik.dtu.dk (D.D.C.); pape@fotonik.dtu.dk (P.M.P.)

**Keywords:** novel food, UVB exposure, pork rind, processing, stability, storage, sensory analysis

## Abstract

Since few foods are naturally rich in vitamin D, novel food products with a high content of vitamin D are needed to decrease the prevalence of vitamin D deficiency. Pork cracklings are Danish snacks with high contents of protein and fat. They are consumed mostly during wintertime when sun exposure cannot fulfil human needs for vitamin D_3_. Pork cracklings were produced in an industrially friendly manner from UVB LED illuminated pork rind, using a combination of sous vide (85 °C, 60 min) and roasting in the oven (200 °C, 20 min). Thermal processing resulted in a significant loss of vitamin D_3_ (>90%). Thus, the process was optimized by the UVB exposure of pork cracklings, i.e., after thermal processing. The produced pork cracklings had a vitamin D_3_ level of ~10 µg/100 g, with a possibility of tailoring its final content. Furthermore, the fat content at 15–20% was a reduction of 50% compared to marketed products in 2021. No significant difference was found in the content of vitamin D_3_ during 31 days of storage in the air. A consumer preference test (*n* = 53) indicated that >80% of participants liked the product and saw its potential as a new food source of vitamin D_3_.

## 1. Introduction

Vitamin D deficiency is a public health problem among the European population. A large observational study estimated that ~40% (*n* = 55,844) of Europeans had vitamin D deficiency [1], defined as the level of 25-hydroxyvitamin D (25(OH)D) in serum being < 50 nmol/L or 20 ng/mL [2]. The prevalence of severe vitamin D deficiency (25(OH)D < 30 nmol/L or 12 ng/mL) reported in Europe was 13%, which is more than in the USA and Canada, but less than in the majority of the world [3]. The adequate intake (AI) for vitamin D by the European Food Safety Authority (EFSA) is set to 15 µg/day [4], whereas the recommended intake (RI) in Nordic countries is 10 µg/day [5]. The average dietary intake reported in national dietary surveys is 3–7 µg/day, which is far from the recommended intake, indicating that the proposed strategy for the fortification of dairy products with vitamin D could not be enough to prevent vitamin D deficiency [6].

Vitamin D is synthesized in the skin by exposure to sunlight, namely, solar ultraviolet B radiation (UVB light) at wavelengths from 290 to 315 nm [7]. It is produced from 7-dehydrocholesterol that converts to previtamin D_3_ upon exposure to UVB light. In the skin, previtamin D_3_ is rapidly converted to vitamin D_3_ in a reversible reaction that reaches equilibrium at 37 °C within 24 h [8]. However, there are several factors affecting the vitamin D production in the skin such as the limited exposure to sunlight due to the use of sunscreens and protective clothes; the high amount of the skin pigment melanin in the epidermis of the skin; and the limited penetration ability of UVB rays to the Earth’s surface above 35° latitude, etc. [9]. A recent descriptive study performed on Danish children and adults (2–69 years) reported substantial seasonal variation in the 25(OH)D concentrations, indicating that 71% of non-supplement users were vitamin D insufficient during at least one of the seasons, mostly during spring [10]. This observation was in agreement with the results of the study that examined the effect of geographic location, season, and month of the year and showed that sun exposure from October until March does not provide vitamin D effective radiation at latitudes >40° [11].

Another major source of vitamin D for humans is food. Vitamin D can be found in foods of animal origin as vitamin D_3_ and vitamin D_2_ and in wild mushrooms as vitamin D_2_. The level of vitamin D in these foodstuffs depends on, among other things, the sunlight and the amount of vitamin D in the feed given to animals [12]. The content of vitamin D_3_ found in Danish free-range pigs was significantly higher in August than in March, indicating that the content of vitamin D found in food is not at a constant level throughout the year but is rather reduced during winter months [12]. Since food of animal origin is mainly produced from animals raised indoors, it is a challenge to meet RI by including fish, meat, eggs, and dairy products in the diet.

To prevent the prevalence of vitamin D deficiency, the European Union (EU) allowed fortification of foodstuffs with vitamin D as long as the added amount is within the recommended range [13]. However, only Sweden, Norway, and Finland produce vitamin D-fortified milk (0.4–2 µg/100 g) as part of either voluntary or mandatory fortification, whereas the rest of EU countries rely on vitamin D intake from natural food sources and supplements [10,14]. There is a number of vitamin D-fortified milk products available on the market in the rest of European countries, but not as part of systematic fortification [14]. It has been stated that the fortification of dairy product is inadequate to ensure the recommended vitamin D through the diet, and there is a need for a wider range of food products with vitamin D [6]. Recently, the EU approved the request for the production and marketing of a novel food product: UVB-exposed fresh mushrooms and mushroom powder with increased content of vitamin D_2_ [15,16]. We have previously demonstrated the production of vitamin D in pork rind by using artificial UVB exposure [17,18]. Thus, we decided to develop a new product, which would have a content of approximately the recommended dietary intake of 10 µg vitamin D in 100 g of the new product [5].

Pork cracklings are a popular snack consumed in Denmark, the US, Spain, and the UK. They are produced by the thermal processing of pork rind and are known as a product of a low nutritional value but are still widely consumed, especially during wintertime. According to the Danish Food Composition Database, pork cracklings contain ~50 g/100 g of protein and ~40 g/100 g of fat [19].

The aim of this study was to develop a process to produce vitamin D enriched pork cracklings with approximately 10 µg vitamin D/100 g which are fat-reduced to a maximum of 20 g fat/100 g, as well as to evaluate stability during the storage and consumer acceptability of the cracklings.

## 2. Materials and Methods

The development and characterization of the vitamin D enriched and fat-reduced pork cracklings was performed in the following steps: optimization of the thermal processing; optimization of the UVB exposure treatment to enhance vitamin D in the pork cracklings; stability study of vitamin D in the pork cracklings during storage; and conduction of a consumer preference test.

### 2.1. Pork Rind

Pork rinds were either purchased as strips of 1 cm width (Danish Crown, Bornholm, Denmark) or cut into 1 cm width strips by a local butcher (Meny, Søborg, Denmark). In any case, the subcutaneous fat was removed from pork rind, and the length of the pork strips was cut to 6.5 cm. However, lengths of 0.5 cm to 3 cm were used in the studies of changes due to heat and storage.

### 2.2. Optimization of the Thermal Processing to Produce Pork Cracklings

For optimization of the thermal processing, pork rind strips were placed on the oven tray, processed in the oven, and salted (1 g NaCl/100 g of pork rind). Seven kinds of thermal processing in the oven using hot air mode (ELK13022-HV, Voss, Denmark) were tested, which varied in time from 30–105 min and in temperature from 100–200 °C. For processing A, two temperatures were used; 100 °C for 60 min, followed by 180 °C for an additional 45 min. Furthermore, as an alternative to cooking, a processing using sous vide was combined with thermal processing in the oven using hot air mode. That processing was named processing B and it included vacuuming (Foodsaver, FFS005X) and processing at a constant temperature by sous vide (85 °C, 60 min; Anova Culinary Sous Vide Precision Cooker, 800 Watts) followed by roasting in the oven in hot air mode (200 °C, 25 min, model ELI14340RF, Voss, Danmark). Table 1 shows the list of the kinds of processing used for optimization.

### 2.3. UVB-Light Source and UVB-Exposure Procedure

A UVB lamp (Prototype LED-295 nm, 60 diodes, 2 mW; DTU Fotonik) with a central wavelength of 295 nm was used. The irradiance (W/nm/m^2^) in the UVB range, i.e., 280–315 nm, from the luminaire was measured using a spectroradiometer (QE65000, Ocean Optics, Duiven, The Netherlands) coupled with a fiber with a Ø1126 µm (BF13LSMA02, Thorlabs, Newton, NJ, USA) attached to a detector probe (CC-3-UV-S cosine diffuser, Ocean Optics, Duiven, The Netherlands). For the experiment, the measured irradiance at each wavelength was multiplied by the factor from the action spectra for the production of vitamin D_3_ from 7-dehydrocholesterol [20]. Thus, the UVB dose in J/m^2^, i.e., the irradiance multiplied with duration of exposure (seconds), was used, thereby considering the differences in the ability of the individual wavelength to produce vitamin D_3_, a dose we suggest be named vitD_3_ dose (vitD_3_ J/m^2^). The characterization of the UVB lamp and the calculation of the parameters for the doses used are described in detail in Appendix A.

The UVB exposure was optimized to identify the dose, i.e., the combination of exposure time and distance between the UVB lamp and the surface of the pork rind that is sufficient to enhance vitamin D_3_ content to ~10 µg/100 g. For each UVB-exposure, one piece of the rind (prepared in Section 2.1) was placed under the middle of the UVB lamp and exposed by the set vitD_3_ dose.

### 2.4. Optimization of the Procedure for Vitamin D Enriched Pork Cracklings

#### 2.4.1. Pre-Experiments

Pork rind was exposed to 10.3, 15.4, and 30.9 vitD_3_ J/m^2^ (*n* = 5), and pork cracklings produced from pork rind (Processing A) were exposed to 15.4, 30.9, and 83.5 vitD_3_ J/m (*n* ≥ 2).

The UVB exposed pork rind strips (Section 2.3) and pork rind strips originating from free-range pigs previously studied [12] were analyzed for the content of vitamin D_3_. Furthermore, vitamin D_3_ content in pork cracklings produced from the above mentioned two types of pork rinds strips was analyzed to examine how the thermal processing affects the vitamin D_3_ content.

#### 2.4.2. Changes in the Content of Vitamin D_3_ during the Thermal Processing to Cracklings

Based on the results from the pre-experiments, we tested the production of vitamin D for pork rind exposed to a dose of 95 vitD_3_ J/m^2^. First, 3 × 3 pork rind strips were UVB-exposed, and each of the nine samples were analyzed for the content of vitamin D_3_.

Then, the UVB dose of 95 vitD_3_ J/m^2^ was exposed to 2 × 16 pork rind prepared for pork cracklings roasted in the oven (ELI14340RF, Voss, Denmark) following processing A and processing B. Thus, for each process, 16 pork rind strips were prepared to study the changes in the content of vitamin D_3_ during thermal processing. The temperature was controlled by a wireless thermometer (Cook and Baker), and the temperature was traceable due to calibration by Testo 735 (Testo, West Chester, PA; ISO17025 accredited). The samples were collected at 8 sampling points (*n* = 2): at 0 min, 15 min, 30 min, 45 min, 60 min, 75 min, 90 min, and 105 min (processing A); and at 0 min, 20 min, 40 min, 60 min, 70 min, 75 min, 80 min, and 85 min (processing B). In order to keep the vitamin D_3_ level within our measuring range (0.5–10 µg/100 g) at all sampling points, the length of the pork rind strips used in this study was between 0.5 to 3 cm.

#### 2.4.3. Pork Rind Thermally Processed to Cracklings and then Exposed to UVB Light

Following the production of pork cracklings produced by procedure B, the cracklings were exposed to a dose of 95 vitD_3_ J/m^2^. The UVB exposure was performed with the skin side up (*n* = 3) and the skin side down (*n* = 3). Furthermore, 3 × 10 pork cracklings with skin side up and down distributed randomly were exposed to the same UVB dose of 95 vitD_3_ J/m^2^ (*n* = 3). After UVB exposure, each group was homogenized (Rommelsbacher EGK 200, Germany) and analyzed in triplicate.

### 2.5. Methods

#### 2.5.1. Vitamin D—Analytical Method

Vitamin D_3_ analysis was performed as previously described [18,21]. In short, 0.2 g of previously homogenized pork cracklings, or 0.3 g of raw pork rind, was weighed together with 0.2 g of sodium ascorbate. ^13^C_5_-labeled vitamin D_3_ (8 ng) was added as the internal standard, and the sample was saponified overnight at room temperature. The vitamin D_3_ was further extracted with 20% ethyl acetate in *n*-heptane by liquid-liquid extraction, cleaned by solid-phase extraction (HybridSPE-phospholipid) and derivatized with 4-phenyl-1,2,4-triazoline-3,5-dione (PTAD) for 5 min in the darkness. The vitamin D_3_ was analyzed by LC-MS/MS [21]. With the exception of methanol used for LC-MS/MS, all chemicals used in the method were of analytical quality.

Quality control was performed using an in-house reference material, Fat 2014, in every batch of samples. The content of vitamin D_3_ in Fat 2014 was 3.84 ± 0.47 ng vitD_3_/g (*n* = 13), which was within the reference range (3.80–4.45 ng/g) and thus confirmed the accuracy of the analysis.

#### 2.5.2. Fat and Dry Matter—Analytical Methods

Fat content was determined according to the Bligh and Dyer method [22]. The fat content was examined in both raw pork rind and processed pork cracklings in two independent groups, with double determination of each group. Half of the raw pork rind from each of the two groups was diced and homogenized, whereas the other half was produced according to processing A and then homogenized by a coffee grinder (Rommelsbacher EGK 200, Dinkelsbühl, Germany). A total of 10 g of the sample was included in the analysis of the fat content, as it was assumed that pork rind and pork cracklings contain >5% fat.

The determination of dry matter was performed in three independent samples of pork rind strips processed by Processing A. Before the analysis, the beakers were weighed and dried for 1–2 h in the oven (Heraeus T5042, Borken, Germany). Approximately 2 g of raw pork rind was weighed and transferred to the previously weighed beakers and reweighed. Beakers containing the samples were kept at 102–105 °C for 24 h. The day after, the samples were left to cool in a desiccator and weighed. The method was based on NMKL, no. 23 ed. 3, 1991 with modifications [23]. Each sample was analyzed in duplicate.

#### 2.5.3. Storage Stability of Vitamin D_3_

150 pieces of vitamin D enriched pork cracklings (1 cm width × 3 cm length) were prepared as described in Section 2.1 and Section 2.4.3. The pork cracklings were homogenized (Rommelsbacher EGK 200, Dinkelsbühl, Germany), and a 1.5 g sample was placed in each of the 24 plastic trays, which then were placed in 24 vacuum bags (Pack marked SR 130 × 260 Polyamid/Polyethylen 90). The experiment included storage stability when pork cracklings were packed in bags filled with pure nitrogen (N_2_) or with air. The packing machine (Multivac, model C500) first vacuumed the sample and then blew N_2_ (12 bags) or air (12 bags) into the bag to a pressure of ~1 atm, followed by the heat-sealing of the bags. The bags were stored in the dark at room temperature (22 °C). On “day 0”, 3 bags each with 1.5 g homogenized samples were placed at −80 °C, and on day 7, day 14, day 31, and day 55, vacuum bags (*n* = 3) were transferred to −80 °C until analysis. Analytical determination was performed five times at day 0, and duplicate for the other days, resulting in 6 analyses per sampling point.

#### 2.5.4. Sensory Evaluation

During the development of the thermal treatment to process pork cracklings, an internal sensory panel consisting of three of the authors performed the evaluation of flavor (no burned flavor after processing), crispiness (easy to bite and chew), and saltiness (appropriate contribution to the flavor). A 5-points hedonic scale from 1 to 5 (best) was used, where the highest total score identified the best thermal processing to produce pork cracklings.

To study the consumers’ liking of the fat-reduced pork cracklings, a consumer preference test was conducted. For the preparation of pork cracklings used in the consumer preference test, 5 different pork rinds were purchased from a local supermarket (Meny, Søborg, Denmark) and prepared as described according to processing A. Freshly prepared pork cracklings were distributed into 100 paper bags packed with air. Testing took place on the streets of Copenhagen, where the bags were distributed to consumers. A questionnaire template was designed in SurveyXact (Rambøll, Copenhagen, Denmark). The questionnaire was composed of 10 questions: (1) What gender do you identify with? (2) Your age? (3) Where did you grow up? Countryside/city? (4) Do you eat pork cracklings? (5) How often do you eat pork cracklings? (6) Did you like the pork cracklings you got? (7) What best describes your experience of the taste, as a whole? (8) What best describes your experience of crispness? (9) What best describes your experience of the salty taste? (10) Other comments? The test evaluated consumers’ liking of the product on a 9-point hedonic scale that ranged from dislike extremely (1) to like extremely (9). Each participant received a package, tasted the product, and evaluated the cracklings by scanning the QR code and answering the questions in the questionnaire. The data were collected in SurveyXact (SurveyXact, Ramboll, Copenhagen, Denmark).

### 2.6. Statistical Analysis

The results are given as the mean ± standard deviation (SD) for the studies with *n* ≥ 3. The correlation between the content of vitamin D_3_ and UVB dose was assessed by Pearson’s correlation coefficient. Pooled t-test was performed to test the significant difference between the stability of pork cracklings produced from UVB-exposed pork rind and from the pork rind originating from free-range pigs. It was also used to test the difference between the packaging in N_2_ and air. One-way ANOVA was used to evaluate significant differences between the days within the same type of packaging (N_2_ or air). Tukey-Kramer HSD test was used to determine the significant difference between different days within the same packaging type. JMP^®^ Pro 15.0.0 (SAS Institute, Cary, NC, USA, 2019) was used for statistical analysis, considering *p* ≤ 0.05 as significantly different.

## 3. Results and Discussion

### 3.1. Optimization of the Thermal Processing to Produce Pork Cracklings

Optimization of the thermal processing of pork cracklings prepared from the pork rind without the presence of subcutaneous fat was performed to develop reproducible production of pork cracklings with the wanted sensory characteristics of flavor (no burned flavor after processing), crispiness (easy to bite and chew), and saltiness (appropriate contribution to the flavor). Three of the authors conducted an internal sensory assessment to evaluate the 8 different kinds of thermal processing (Table 1). The maximum score was obtained by processing A and processing B. In short, processing A included roasting in the oven at a low temperature (100 °C, 60 min), followed by roasting at an increased temperature (180 °C, 45 min). Processing A was performed by combining two different temperatures, where the 100 °C simulated the boiling procedure in the traditional production of pork crackling, where pork rind is initially cooked in boiling water, which is followed by treatment in the oven. For processing B, the first part of the oven treatment was replaced by a sous vide that included initial processing of the pork rind by sous vide (85 °C, 60 min) followed by roasting in the oven (200 °C, 25 min). Sous vide is a processing method widely used in industrial food production that enables precise control of the temperature and time during processing and at the same time improves the food textural, nutritional, and sensory quality compared to traditional cooking [24,25]. It is used as a pre-preparation of the product that can easily be finished in the short time right before consumption, providing the product with adequate crispiness and freshness of the flavor. Besides, the use of processing B enabled shorter roasting in the oven. We did not find it essential for the new vitamin D enriched and fat-reduced product to conduct further optimization of the thermal treatment, e.g., by combining the first part of processing A with last part of processing B and vice versa.

Due to the very similar products achieved by thermal processing A and thermal processing B, we used the two treatments interchangeably throughout the study. However, in the investigation of the changes in content of vitamin D during the thermal treatment, both treatments were investigated.

### 3.2. Optimization of the Procedure for Vitamin D Enriched Pork Cracklings

#### 3.2.1. Pre-Experiment

**Figure 1:**
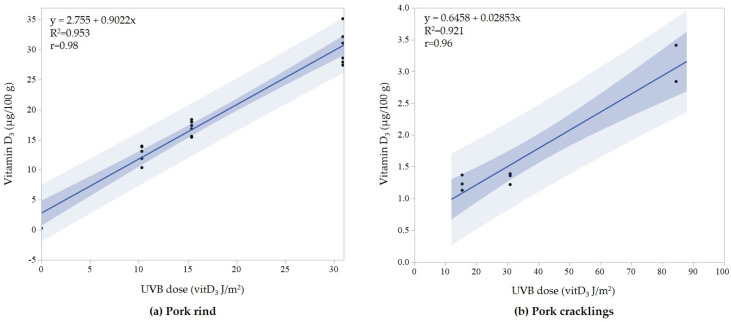
The production of vitamin D_3_ related to the exposed UVB dose. (**a**) Vitamin D_3_ (μg/100 g) produced in raw pork rind at UVB doses of 10, 15, and 31 vitD_3_ J/m^2^; (**b**) Vitamin D_3_ (μg/100 g) produced in pork cracklings produced from pork rind UVB exposed by 15, 31, and 84 vitD_3_ J/m^2^. Dark shade represents the confidence interval for the fitted line, while light shade represents a confidence interval for individual values (*p* ≤ 0.05).

The pre-experiment (Section 2.4.1) firstly examined the exposure of pork rind to three different doses: 10 vitD_3_ J/m^2^, 15 vitD_3_ J/m^2^, and 31 vitD_3_ J/m^2^. Figure 1a presents a linear relationship between the content of vitamin D_3_ and UVB exposure dose in UVB-exposed pork rind. Pearson’s correlation coefficient was 0.98, indicating a strong positive relationship between vitamin D_3_ produced in the pork rind and the UVB dose used. Secondly, vitamin D in pork cracklings was produced from UVB-exposed pork rind. The UVB doses used were 15, 31, and 84 vitD_3_ J/m^2^ (Figure 1b). In this case, Pearson’s correlation coefficient was 0.96, again indicating a strong positive relationship between vitamin D_3_ in pork cracklings and the UVB dose used. Remarkable is the low content in the cracklings compared to the rind, which is equivalent to a loss >90%.

The correlation between vitamin D_3_ production and UVB dose was investigated by Barnkob et al. in pork rind [17]. They studied the content of vitamin D_3_ at 6 different doses (300–20,000 J/m^2^) at a wavelength of 296 nm. The data showed linear correlation followed by a logarithmic model, resulting in high concentrations of vitamin D_3_ (3.5–4 µg/cm^2^). MacLaughlin et al. [26] UVB-exposed human skin to very high doses (10,000–300,000 J/m^2^) at a wavelength of 295 nm, which also followed a linear correlation at lower doses but turned to a logarithmic model at higher levels. We used doses in the linear interval, and our results showed that it is possible to expose pork rind to a specific UVB dose by changing the time and distance and to achieve the tailored concentration of vitamin D_3_; but they also showed that there is a vast loss of vitamin D_3_ during the thermal processing of crackling.

As the loss of vitamin D_3_ after thermal processing was unexpected, we decided to test the importance of the origin of vitamin D_3_ in pork rind, i.e., if the production of vitamin D_3_ while pigs were alive or after they have been slaughtered affected its stability in the final product. Thus, pork cracklings were prepared from the pork rind originating from free-range pigs (see Section 2.4.1). Vitamin D_3_ content in the raw pork rind ranged from 1.5–2.5 µg/100 g. After processing, all (99 ± 1%) vitamin D_3_ was lost, even though it was produced while pigs were alive, which leads us to the conclusion that the temperature could be responsible for the vitamin D_3_ loss.

#### 3.2.2. Changes in the Content of Vitamin D_3_ during the Thermal Processing to Cracklings

In order to optimize the production of pork cracklings with tailored vitamin D_3_ content, we decided to study the stage of thermal processing at which the loss of vitamin D_3_ occurs. For the experiments, we chose a slightly higher UVB-dose, i.e., 95 vitD_3_ J/m^2^, which was initially shown to provide 61 ± 6 µg vitamin D_3_/100 g. Thus, our method would be able to quantify a loss of 99%, i.e., 0.6 µg/100 g.

**Figure 2:**
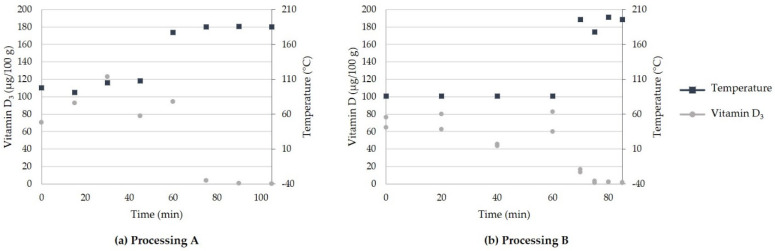
Stability of vitamin D_3_ during the thermal processing of UVB-exposed (95 vitD_3_ J/m^2^) pork rind. (**a**) Processing A: Roasting in the oven under hot air in 2 intervals (100 °C, 60 min and 180 °C, 45 min). (**b**) Processing B: Sous vide (85 °C, 60 min) followed by roasting in the oven (200 °C, 25 min). Vitamin D_3_ (µg/100 g) content reads from the left *y*-axis, while the temperature reads from the right *y*-axis. The values for each single determination at each sampling point are presented.

Figure 2 presents the results from the study described in Section 2.4.2, showing that, for both treatments, >90% of the vitamin D_3_ was lost.

The results indicate a decreased stability of vitamin D_3_ at temperatures >100 °C. A slight increase in the vitamin D_3_ content during processing was related to the weight loss during the increase in the temperature.

Previous studies on the stability of vitamin D_3_ in food during thermal processing indicated that a loss <50% could occur; thus, the observed loss >90% was unexpected. Bennink and Ono observed a vitamin D_3_ loss of 35–42% in beef when examining broiling, roasting, and braising, where the highest loss was observed during braising [27]. Contrary to our results, Clausen et al. reported a loss of vitamin D_3_ in processed pork rind at ~26% [28]. However, their processing included the roasting of pork at 250 °C for 20 min followed by 150 °C until the meat core reached 80 °C. Vitamin D_3_ stability studies performed in a model system by adding vitamin D_3_ to the sunflower oil processed by household cooking procedures such as pan-frying, microwave, boiling, and roasting at different times and temperatures in the oven indicated a relatively little loss of 1–30% [29]. Goebel et al. studied the stability of vitamin D_3_ added to canola oil during baking of a dark chocolate brownie at 80–230 °C for 10–40 min by using various antioxidants [30]. They reported a vitamin D_3_ loss of up to 90% when exposed to temperatures of 180–230 °C, even with the presence of antioxidants. In other foodstuffs such as eggs, margarine, bread, and rainbow trout, the loss was in general <30%, except for eggs and margarine heated in the oven at ~160 °C for 40 min, which resulted in a loss of ~60% [29,31]. This indicates that cooking procedures (especially processing in the oven), vitamin D_3_ content, and the type of food matrix play a significant role in the stability of vitamin D_3_. Thus, the stability of vitamin D during thermal processing is affected to a high extent by temperature and not oxidation.

#### 3.2.3. Pork Rind Thermally Processed to Cracklings and then Exposed to UVB Light

The dose of 95 vitD_3_ J/m^2^ enabled the production of 9.0 ± 2.8 µg vitamin D_3_ per 100 g of pork cracklings, with no significant difference between the pork cracklings UVB-exposed on skin side up or skin side down (*p* = 0.265). It is possible to produce vitamin D_3_ in pork cracklings, but it is 15% of the amount produced by a similar vitD_3_ dose exposed to pork rind. However, the dose of 95 vitD_3_ J/m^2^ produced the acceptable amount of ~10 vitD_3_/100 g.

Table 2 shows the results for the content of vitamin D_3_ when 30 pork cracklings were divided into 3 groups of 10 pork cracklings each (Exposed group 1–3). Each group was UVB exposed with a dose of 95 vitD_3_ J/m^2^.

Exposed group 2 was significantly different than the other two groups (*p* < 0.001), as less vitamin D_3_ was produced during the UVB exposure of this group. This result indicates that the variation between the separate UVB exposures in our set-up might be too high or that 10 pork cracklings are too few to use to test homogenization.

### 3.3. Characterization of the Vitamin D Enriched and Low-Fat Pork Cracklings

#### 3.3.1. Vitamin D

In the development of a vitamin D enriched pork cracklings, we have shown that vitamin D can be produced in pork cracklings by UVB-exposure. The dose of 95 vitD_3_ J/m^2^ will provide a content of approximately 10 µg vitamin D/100 g. The observed variation indicates that, for an industrial production, the validation of the UVB-exposure should ensure that the variation is acceptable for the declaration of vitamin D.

#### 3.3.2. Fat and Dry Matter Content

The pork rind used for the cracklings contained 15 ± 4.6% (*n* = 4) of fat, whereas the pork cracklings produced by processing A contained 17 ± 4.1% (*n* = 4) fat content. This is a 50% reduction of fat in the pork cracklings compared to pork cracklings on the Danish market, which contain ~40% fat [19]. The content of dry matter was 41 ± 2.6% (*n* = 6), of which the major part is protein, as stated in the Danish Food Composition Database [19].

#### 3.3.3. Storage Stability

The findings from Section 3.2.3. led us to a decision to use homogenized pork cracklings for the study of storage stability. The samples were prepared by processing B, as described in Section 2.4.3. The results of the 55 days storage study are shown in Table 3.

There is a significant difference in the stability of vitamin D_3_ between the storage of pork cracklings in N_2_ or air (*p* = 0.009). Vitamin D_3_ was stable during 55 days of storage in the packaging containing air, whereas a significant loss (~18%) was observed after 31 days of storage in N_2_ (Table 3). Our results are in agreement with the previous study that examined vitamin D_3_ stability during 70 days of storage of fortified canola oil [32]. No significant difference was found when canola oil fortified with vitamin D_3_ was stored at 27 °C with and without air in the darkness, resulting in loss of vitamin D_3_ of ~10% after 55 days of storage. It was interesting to see that vitamin D_3_ is less stable if stored in N_2_ than air, as N_2_ is often used when storing vitamin D_3_ to prevent oxidation. Based on these results, packaging of the pork cracklings in air will ensure the content of vitamin D_3_ to be similar after one month of storage as on the day of packaging. However, the limitations in our storage stability study are the fact that the design included the only three samples at each sampling point and that we observed an unexpected increase in vitamin D from day 0 to day 7.

#### 3.3.4. Sensory Evaluation

Pork rind strips used in this step were not exposed to UVB light, as it was assumed that UVB exposure will not affect the taste and texture of cracklings.

**Figure 3:**
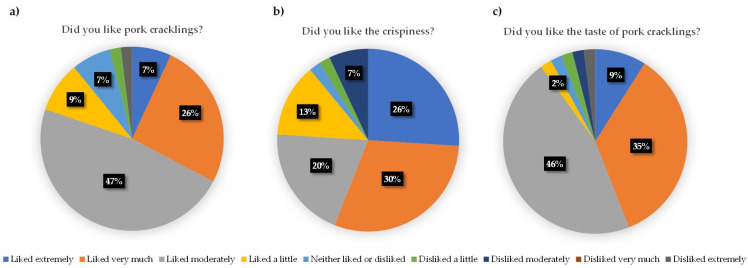
Results of the consumer preference test (*n* = 55). (**a**) Consumers’ opinions about the product. (**b**) Consumers’ opinion about the crispiness of the product. (**c**) Consumers’ opinion about the taste of the product.

In order to find out if the consumer would like the product, we performed a consumer preference test. The aim was to include 100 consumers, but due to the restrictions related to spreading the COVID-19 virus, we could not reach our target number of participants. A total of 55 people participated in the evaluation of the product; 53 completed and 2 partially completed the survey. Since the consumer was not recruited and the distribution of gender among the consumers that participated was in balance, it shows that the target population for the product would be both women (51%) and men (49%). A total of 47 (85%) participants answered that they usually eat pork cracklings, which indicated that they were able to evaluate the characteristics of the product, as they had some preference in product characteristics. The results of the product characteristics are presented in Figure 3.

As shown in Figure 3a, >80% of participants answered that they liked the product. Some of them would prefer to have more fat in the product, which is understandable, as pork cracklings are known as a product where fat plays an important role in the final flavor. Figure 3b presents the consumers’ opinions on the crispiness, and, even though 89% of participants liked the crispiness, some of them commented that pork cracklings were a bit hard, which indicates that there is variability in the production. It has to be mentioned that sous vide optimization was performed after the consumer preference test, and the use of that processing in the first step of the production of pork cracklings enables reproducibility when it comes to crispiness. In general, >80% of participants liked the taste and texture of the produced pork cracklings, indicating the potential of this product on the market.

The overall aim of the consumer preference test was to evaluate consumers’ liking of the product and to collect any new suggestions from potential customers in order to improve the product. Although the responses would be affected by several factors, such as the consumer’s lifestyle, mood, nutritional knowledge, interest in health issues, and age, they give the overall impression of consumers liking the product. However, we did not examine how the idea of bio-fortification of pork cracklings by vitamin D_3_ would affect consumers’ satisfaction. Food fortification with vitamin D is often performed in dairy products, and it is voluntary in Europe [14]. However, it is still controversial among the Danish population, which is supported by the results from a previous study on consumer acceptance of food fortification by vitamin D, where only 6% had a positive attitude vs. 38% that had a negative attitude towards food fortification in general [33]. When it comes to fortification with vitamin D, 27% of participants had a positive attitude, 28% had a negative attitude, and 45% were neutral. This increase in acceptance is probably the result of the general awareness about vitamin D deficiency that occurs in Denmark. Furthermore, vitamin D_3_ is produced from 7-dehydrocholesterol naturally found in pork rind upon UVB exposure. Thus, this product is not classified as an enriched product, and it is important to inform the consumer about that. The newly developed product may be labeled as “a source of vitamin D” or as “high in vitamin D content”, as it is possible to tailor its content and increase it until it contributes to at least 30% of the recommended intake [5,34].

## 4. Conclusions

It is possible to produce a new food product with a high consumer liking: pork cracklings with enhanced vitamin D_3_ content and reduced content of fat in an industrially friendly manner using the combination of sous vide and roasting in the oven or the traditional combination of two temperatures in the oven. A significant loss of vitamin D_3_ (>90%) observed during the thermal processing of pork cracklings at temperatures >100 °C was overcome by the UVB exposure of the thermally processed product. The use of UVB exposure of pork cracklings made it easy to tailor the content of vitamin D_3_. This can be combined with the tailored size and shape of the product to meet consumer preferences. Thus, consumption of this product could substantially contribute to the recommended daily intake of vitamin D (10 µg/100 g). Pork cracklings contained ~50% less fat content than pork cracklings originally found on the market and were stable for 31 days if packed in air and stored in the darkness at room temperature. Sensory evaluation showed that >80% of participants liked the new product and that there is a potential for its release as a novel food with a nutrition claim “high in vitamin D”.

## Figures and Tables

**Table 1:** Parameters used in the optimization of the thermal processing from rind to cracklings.

Processing	Sous Vide		Oven with Hot Air Mode		Total Time (min)
	Temperature (°C)	Time (min)	Temperature (°C)	Time (min)	
1	-	-	180	60	60
2	-	-	190	40	40
3	-	-	190	45	45
4	-	-	200	30	30
5	-	-	200	40	40
6	-	-	200	50	50
A	-	-	100 + 180	60 + 45	105
B	85	60	200	25	85

-—Sous vide not used.

**Table 2:** Reproducibility of UVB exposure after thermal processing B. Each exposed group consisted of 10 pork cracklings, each analyzed in triplicate. The content of vitamin D_3_ is shown as mean ± SD.

Exposed Group	Vitamin D_3_ (µg/100 g)
1	9.3 ± 0.5 ^a^
2	5.5 ± 0.6 ^b^
3	10 ± 0.8 ^a^

A different letter (a, b) next to the value indicates a significant difference (*p* ≤ 0.05).

**Table 3:** Stability of vitamin D_3_ during 55 days of storage in a controlled atmosphere of N_2_ or air. The values are presented as mean ± SD.

Days	Replicates (*n*)	Vitamin D_3_ (µg/100 g)	
		N_2_	Air
0	5	13 ± 0.4 ^b^	13 ± 0.4 ^a,b^
7	6	15 ± 0.8 ^a^	15 ± 1.4 ^a^
14	6	14 ± 0.7 ^ab^	15 ± 0.8 ^a^
31	6	12 ± 0.6 ^c^	14 ± 1.0 ^ab^
55	6	11 ± 0.4 ^d^	13 ± 0.7 ^b^

A different letter (a–d) next to the value indicates a significant difference between different days within the same type of packaging (*p* ≤ 0.05).

**Figure A1:**
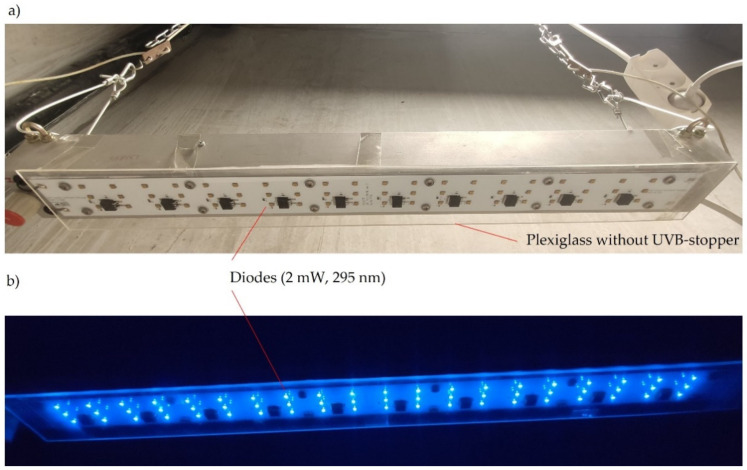
The LED-UVB 295 luminaire turned off (**a**) and turned on (**b**).

**Figure A2:**
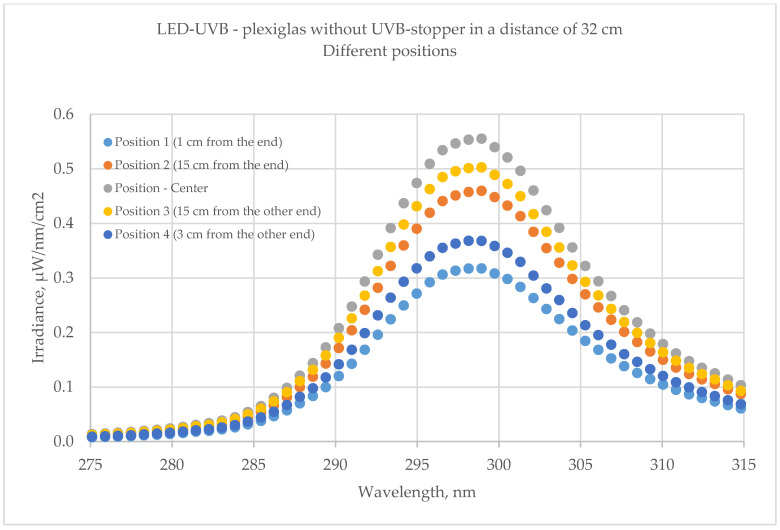
Irradiance (275–315 nm) of the LED-UVB 295 luminaire at a distance of 32 cm.

**Figure A3:**
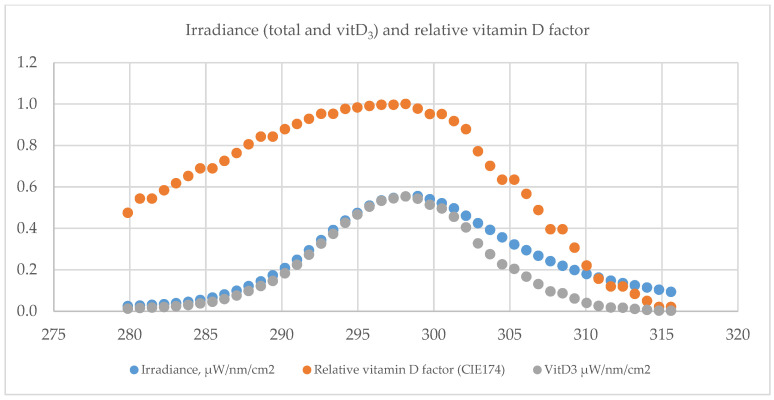
Illustration of the total irradiance, the vitamin D producing irradiance, and the relative factor for the conversion of 7-dehydrocholesterol to vitamin D_3_ at each UVB-wavelengths.

## Data Availability

The data presented in this study are available on request from the corresponding author.

## Appendix A

The UVB-lamp (LED-UVB 295) consisted of 60 diodes (Dongguan Hongke Lighting Co., LT, Dongguan, China), each 2 mW with 295 nm as the main wavelength. Plexiglass without a UVB-stopper was mounted for safety reasons (Figure A1).

The irradiance (W/nm/m^2^) from the luminaire was measured using a spectroradiometer (QE65000, Ocean Optics) fiber coupled with a fiber with a Ø1126 µm (BF13LSMA02, Thorlabs) attached to a detector probe (CC-3-UV-S cosine diffuser, Ocean Optics). The detector probe was aligned perpendicular at a distance of 32 cm from the luminaire, and measurements was done at five different positions on every 0.8 nm. The irradiance of the LED-UVB 295 luminaire can be seen in Figure A2.

The vitamin D producing capacity of the LED-UVB 295 luminaire was calculated by using the individual factors for the conversion of 7-dehydrocholesterol to vitamin D_3_ [20], i.e., at each wavelength, the irradiance was multiplied by the CIE-factor. The results are illustrated in Figure A3.

Then, the dose (vitD_3_ J/m^2^) was calculated by the multiplication of the vitD_3_ irradiance (vitD_3_ W/m^2^) with the duration of the exposure (seconds).

If a distance for the UVB-exposure differed from 32 cm, the irradiance at 32 cm and at the chosen distance, e.g., 10 cm, was measured by a handheld UVB-photometer (ILT 1400-BL, International Light Technologies, Peabody, MA, USA). The irradiance at 32 cm was multiplied with the relative difference measured by the handheld photometer, e.g., at 32 cm and 10 cm, the UVB-photometer measures 200 µW/cm^2^ and 37 µW/cm^2^, respectively. Thus, the irradiance is 200/37 times higher at 10 cm.

In the experiment, the distances used were 32 and 10 cm, and the duration of exposure was 140 s to 420 s.
